# A mobile robot bridging manual and automated bioscientific workflows by applying the Swiss army knife principle

**DOI:** 10.1038/s41598-025-05404-3

**Published:** 2025-06-20

**Authors:** Nicole Rupp, Rebecca Wienbruch, Julia Esther Gröber, Michael Köppl, Ruven Dreischke, Larissa Ballardt, Klaus König, Thole Zuchner

**Affiliations:** 1 Faculty for Life Sciences, Professorship for Bioanalytics and Laboratory Automation, Albstadt-Sigmaringen University, Anton-Günther-Str. 51, 72488 Sigmaringen, Germany; 2 8-BOT Robotics, Schiffstr. 46, 78464 Konstanz, Germany; 3 jetzt-GmbH, Bücklestrasse 3, 78467 Konstanz, Germany

**Keywords:** Laboratory automation, Camera-based, Quality improvement, Bioanalytical processes, Multifunctional, Robotic system, Computational platforms and environments, Data processing, Hardware and infrastructure, Image processing, Machine learning, Software

## Abstract

The complexity and diversity of bioscientific research laboratories, creates significant challenges for automation. Their varying workflows, personnel, and instruments, often hinder smaller research laboratories to benefit from automated processes, as existing systems seem unsuitable due to low flexibility. Therefore, we developed a versatile robotic system designed to automate a broad range of bioscience laboratory processes. Central to our system and novel, compared to all other kinds of laboratory automation concepts, is a multifunctional end effector, inspired by the Swiss-army-knife, capable of executing multiple tasks, including an operating finger, a camera system, a gripper, and a pipette. This end effector is mounted on a 6-axis robotic arm, supported by a mobile base, enabling easy transport across different bioanalytical laboratory environments. Utilizing windows manipulating scripting routines, allows the automation of diverse software programs including software-based laboratory devices. We demonstrate the capabilities of the Laboratory Automation Robotic System (LARS) by automating the pH buffer adjustments, showcasing its potential to improve efficiency and reproducibility in bioscience research. The resulting prototype allows the integration of any laboratory instrument into a desired automation routine without limitations concerning device interfaces, while using a highly flexible multifunctional end-effector as a replacement of the human hand and eye.

**Supplementary Information:**

The online version contains supplementary material available at 10.1038/s41598-025-05404-3.

## Introduction

The positive impact associated with laboratory automation in biosciences has been described many times^1–3^. Especially on large scale (e. g., clinic), laboratory automation is well established and has essentially contributed to efficiency, cost savings and quality improvements^3–5^. However, automating bioscientific processes in smaller laboratories—including academic research—is still challenging^6^. Here, processes are highly diverse and often have shorter life cycles, compared to those in industrial environments, which makes a standardization of processes difficult^4^.

Diverse commercial suitable systems for these kind of laboratories are available, but represent usually stand-alone systems, automating just a single process step^7,8^. With the variety of systems on the market, the choice of which system to purchase is complex: Automation systems need to be carefully considered according to the needs of the individual laboratory in order to deliver the promised success^9,10^. In addition, laboratory professionals frequently find themselves tackling technical aspects such as installation, commissioning, maintenance, and troubleshooting alone^2^. This degree of responsibility often contributes to laboratory operators’ reluctance to embrace automation^9^. However, smaller laboratories are still under pressure to improve quality, especially in bioanalytics, while costs, laboratory space and resources are strictly limited^11^. Therefore, a flexible automation system that is portable and capable of operating existing laboratory equipment automatically would be highly desirable for those environments^12,13^.

Automating devices that have been specifically designed for human users might not seem trivial^14^. A growing number of system integrators on the market offers the opportunity to automate processes integrating existing devices in any laboratory in combination with newer technology (e.g., robots). Still, these kind of tailor-made automations are usually time-consuming in development, cost-intensive and often not flexible enough to allow greater changes when processes evolve^15^. Furthermore, the manual operation of an integrated device may be constrained when it is required for another manual task. These difficulties are pushing the field of “do-it-yourself” solutions^7^.

However, this poses the challenge of interface issues that frequently arise when integrating different devices^13^. Over the years, a laboratory evolves in terms of processes and equipment. Consequently, such laboratories often feature a mix of modern (automated) instruments alongside older devices that have been functioning effectively for several decades^16^. The Standardization in Laboratory Automation (SiLA) tries to establish a standard for better interaction and integration beyond the boundaries of device manufacturers. Nevertheless, not all manufacturers have yet accepted the need for an uniform standard^14^. In addition, existing and sometimes old devices have to be upgraded to support SiLA, which results in an effort of time, money and resources^17^. In a dynamic environment such as smaller laboratories, however, the total migration of all devices to automation is highly questionable and not initially target-oriented^9^.

In this study, we present an approach of automation that is highly flexible and suitable for smaller and medium sized (research) laboratories. Our strategy aims to automate any bio-scientific laboratory as it currently operates, without requiring major alterations to the existing manual workflow and without the requirement to replace existing, manual laboratory instruments. Our aim is to offer the target laboratories a suitable opportunity to improve processes through automation to improve the reproducibility and overall quality of experimental data. For this, we developed an automation system as a prototype consisting of a mobile platform, a robotic arm operating a multifunctional tool and a Windows software automation environment. This system—called LARS (Laboratory Automated Robotic System)—is designed to operate laboratory equipment like a manual user and therefore has no limitations in terms of different device interfaces. This includes both, hardware and software-based tasks. Our goal has been to enable LARS to perform a wide range of tasks within a bioscientific laboratory. To achieve this, we equipped the system with multifunctional capabilities reminiscent of a Swiss-army-knife, allowing it to execute various functions without requiring retooling. Thus, LARS is capable to transport microtitre plates, to operate buttons and switches on devices, to handle liquids (e.g., in the range of 10–200 µL) and to read-out displays. The idea of a multifunctional tool is not new and has already been applied in tailor-made laboratory automation solutions and stand-alone systems. However, to the best of our knowledge, it has not yet been developed into a universal tool suitable for use with any robotic system and any laboratory process. As LARS is a mobile system, laboratory operators can deploy it across different laboratories and on various equipment, or store it when not in use. We see numerous advantages in the strategy of LARS: as it comprises a single system, laboratory personnel only need to be trained for one system to enable multiple automations. Moreover, LARS utilizes existing equipment, facilitating the successful transfer of methods to the automation system. LARS minimizes investment risks by automating various processes, provides laboratory operators the opportunity to experiment with automation, and ensures flexibility to adapt to evolving processes.

As LARS is in the prototype phase, it provides opportunities for further refinement and expansion. In this paper, we present LARS as a strategy and potential for the efficient and successful automation of a smaller, highly variable laboratory environment. This strategy leverages existing technologies, such as camera-based positioning of robots and multifunctional end effectors, to create a novel system. It represents one of the first automation strategies in laboratory automation where these known components are combined specifically to meet the needs of smaller research laboratories.

## Materials and Methods

### LARS

For LARS, we developed a strategy to allow the interaction between diverse hardware and software components. As main components of LARS, we utilized the 6-axis robot Horst 600 (fruitcore robotics GmbH, Konstanz, Germany) for physical tasks in the laboratory. The corresponding software HorstFX (version 2022.07_hotfix6) runs on Linux (Debian GNU/Linux 10 (buster)). The manufacturer provides the complete system as ready-to-use. As mobile basis, we utilized a hydraulic trolley (KAISER + KRAFT GmbH, Stuttgart, Germany). For further constructions (e.g., the mobile platform), we used aluminum profiles and stainless-steel components, as well as resopal plates. For the multifunctional end effector, we modified 8-BOT’s labhand (gripper for microtiter plates) using mostly 3D printing (Polylactide). Further, we have made use of components such as an electronic pipette (200 μL Transferpette, BRAND GmbH & Co. KG, Wertheim, Germany) and a Raspberry Pi Camera 3 (Raspberry Pi Foundation, Cambridge, UK). We also utilized ArUco markers (25 × 25 mm), generated them online (https://fodi.github.io/arucosheetgen/) and printed them in original size. To control the end effectors functions, we used a Raspberry Pi Zero 2 W (Raspberry Pi Foundation, Cambridge, UK), and Raspberry Pi PiIO DIO HZ digital IO board (Raspberry Pi Foundation, Cambridge, UK) with wireless location area network (WLAN). On a Windows PC (Intel Core i7-6600U CPU, 8 GB RAM, Windows 10 Enterprise, Microsoft, Redmond, Washington) we installed a Virtual Network Computing (VNC) tool (TightVNC Viewer Version 2.6.63, GlavSoft LL.C., Tomsk, Russia) and AutoIt version 3.3.14.5 (AutoIt Consulting Ltd., Worcestershire, UK) with the corresponding AutoIt Script Editor (SciTE4AutoIt3, Neil Hodgson) version 4.4.6 for the automated software processes.

### Experimental Application

To demonstrate LARS’s capabilities, we aimed to automate a complex laboratory process using multiple functionalities of the end effector. We identified the buffer pH value adjustment process as suitable for this demonstration, as it is a frequent and sometimes time-consuming process in our institute and as such involves numerous steps, such as switching on devices, gripping and transferring objects, pipetting, reading of optical displays, decisions about the volumes to be pipetted and documentation of data. Of course, different automated setups for pH adjustment already exist. However, with LARS, there is no need to purchase such a system and we chose this task solely to demonstrate the various capabilities of the system. We show the whole automated process in a video (Supplementary video S1).

For automated pH value adjustment, we developed a pH prediction tool, that is rooted on regression formulas of titration curves. In detail, we utilized three HCl (hydrochloric acid 32%, Carl Roth, Karlsruhe, Germany) concentrations (c1 = 2.5 mol/L, c2 = 5 mol/L, c3 = 10 mol/L) and three NaOH (Sodium hydroxide solution 40%, Carl Roth, Karlsruhe, Germany) concentrations (c1 = 2.5 mol/L, c2 = 5 mol/L, c3 = 10 mol/L) diluted with dH_2_O. We selected data for titration curves for each concentration by adding a specific volume stepwise to phosphate-buffered saline (PBS) (1 L volume) around pH 6 and 8. The buffer was constantly stirred by a magnetic stirrer (RSM-10HP, Phoenix Instrument, Garbsen, Germany). For pH measurement, we utilized the pH 50 + DHS pH-meter (XSinstruments Carpi MO, Italy). We used the devices for both, manual development of the prediction tool and for the automated process with LARS. To achieve the titration curve, we plotted the volume change against the corresponding pH change, resulting in six independent curves and their regression formula.

**Fig. 1:**
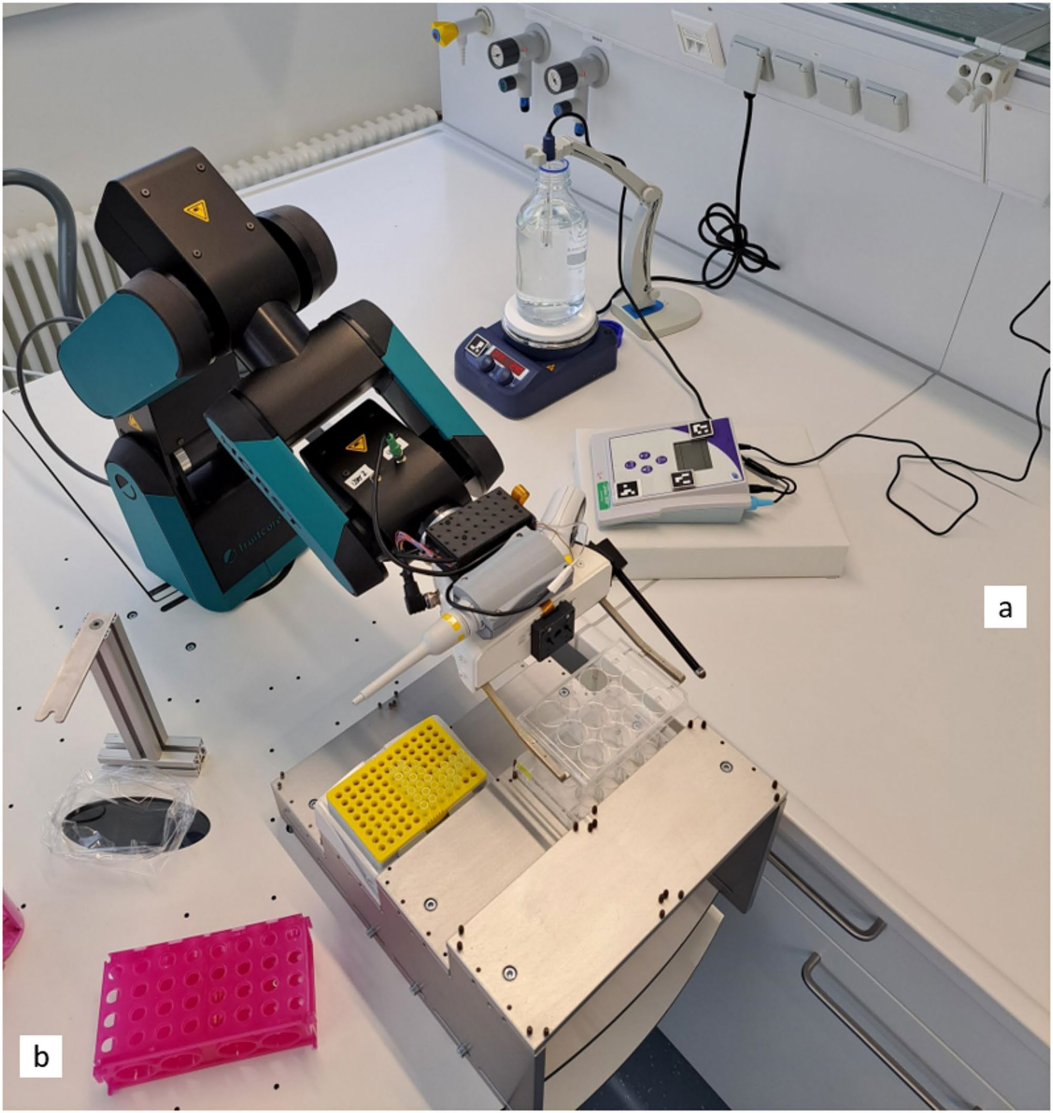
Setup of automated pH adjustment using LARS. The utilized devices—magnetic stirrer and the pH meter—are placed on the laboratory desk **a**. The buffer is placed on the magnetic stirrer with the immersed pH electrode. One unique ArUco marker on both devices is needed for device detection; two additional markers on the pH meter (next to the display) are required for display read-out. The acid–base library (12-well plate) is placed on the stair hotel on LARS’s platform **b**. For pipette tip handling, the stripping station and a tip box is used (platform).

For the laboratory setup, we placed LARS in front of a laboratory desk and positioned the pH meter and the magnetic stirrer—each with individual ArUco markers—within the robot’s range of movement. Initially, the buffer is placed in a 1 L glass bottle (without screw cap) on the magnetic stirrer and the pH sensor is placed in the buffer for continuous measurement. We provided a library of the determined HCl and NaOH dilutions in a 12-well plate. The library 12-well plate and the tip box are placed on the stair hotel on the LARS platform. The setup of this automation is shown in Fig. 1.

## Results and discussion

### LARS

**Fig. 2:**
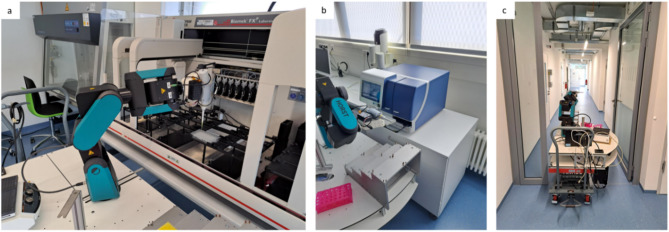
Flexibility of LARS. **a**. LARS is capable to interact with liquid handling systems. As those automated machines have their strength in the rapid dispensing of entire microtiter plates, LARS is able to operate individual wells particularly reliably with a single-channel pipette. **b.** LARS feeds a spectrometer with a previously prepared microtiter plate. **c.** Mobile system: Due to the mobile platform, LARS can be easily transported between laboratories.

LARS consists of three parts: first the mobile platform, second the robotic arm and its end effector and third, the automated user based on the software package AutoIt. Therefore, LARS is manifold flexible. First, the Swiss-Army-Knife-like end effector is able to fulfil various tasks in any laboratory. Second, the mobile platform allows an easy movement of LARS between laboratory desks or laboratories and third, any Windows software can be integrated into automation using AutoIt. Figure 2 shows examples on how simple LARS can be applied in various laboratory situations.

### Experimental Example

**Fig. 3:**
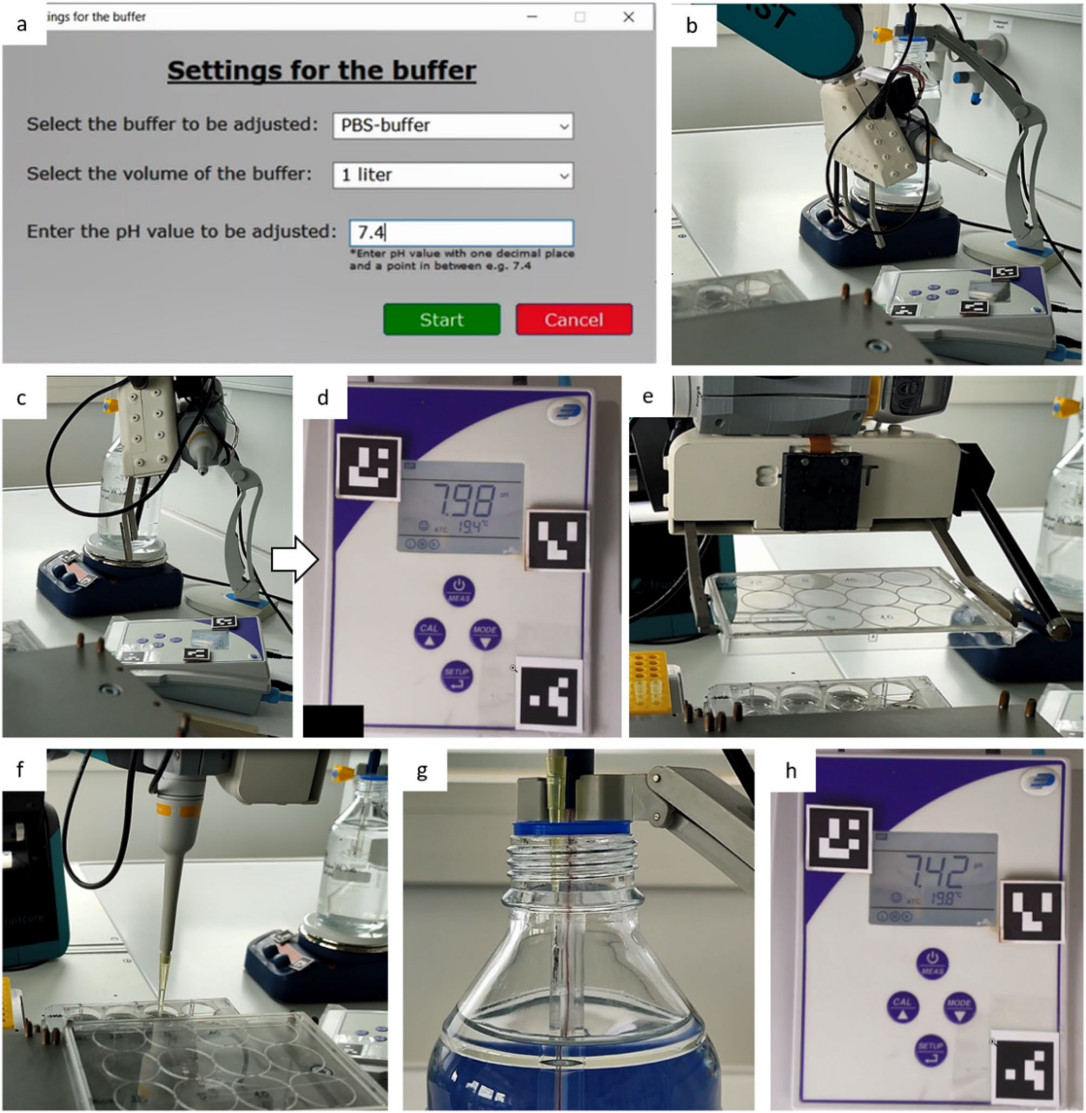
Excerpts from Supplementary video S1 showing the automated pH value adjustment. After starting the process, the user needs to provide information via a graphical user interface **a**. The robot uses the operating finger to turn on the devices by pressing the corresponding physical buttons **b**. To detect the current pH value, the pH meters display is photographed by the camera **c,** while the image is displayed on the Windows PC screen **d**. The robot opens the acid/base-library with the gripper **e** and aspirates the corresponding liquid calculated by AutoIt’s prediction tool **f.** After dispensing into the buffer **g**, the process is repeated until the target value is reached **h**.

Figure 3 shows excerpts from our Supplementary video S1 illustrating the automated pH adjustment process. To fulfil the automated process, we utilized several functions of the LARS end effector: operating finger to switch on both devices by pressing the corresponding button (Fig. 3b), camera for device detection and display read-out^18^ (Fig. 3c-d), plate gripper to open and close the library lid and the pipette for aspirating and dispensing a liquid (acid or base). At the beginning of the automated process, we provide an AutoIt based graphical user interface (GUI) where the user is able to enter parameters that are necessary for pH value prediction (Fig. 3a).

To achieve the target pH value, we developed a pH prediction tool, based on the titration curve formulas described in the material and method section. We integrated the formulas into an AutoIt script to calculate the needed volume of a specific concentration by inserting the target and the actual pH value into the formula. The target pH of 7.4 is set via GUI in AutoIt (Fig. 3a), while the actual pH value is displayed by the pH meter. To get the actual pH value, the camera takes an image of the pH meter’s display (Fig. 3c). Then, a python-based display recognition tool^18^ analyses the image and stores the actual pH value in a csv file, where AutoIt is able to process it for the pH prediction. As result of the prediction tool, AutoIt decides whether to aspirate an acid or a base (Fig. 3f) and in which concentration to achieve the desired target pH value. After the pipetting step (Fig. 3g), LARS analyses again the pH meter’s display to compare the actual pH value with the target pH value (Fig. 3h). If the discrepancy is > 0.05, the procedure will be repeated. For example, in the case shown in our video, it took three pipetting rounds to achieve the target pH value.

Our prediction tool is capable to handle PBS buffers in pH value range between 6–8. The system allows further extensions (e.g., other buffer, volume or target pH) if applicable. In the end, the user receives a protocol, where the automations steps are traced. For example, the protocol provides the initial pH value, added liquid, all intermediate pH values (in case of several pipetting steps) and the final volume. AutoIt also saves all generated raw data (camera images of pH meter display).

The automation described here exemplifies one of the more intricate automated processes. Rather than simply replicating manual procedures, LARS must be equipped to make complex decisions (chose a dilution to achieve the target pH value). This necessitated the development and implementation of the prediction tool incorporating mathematical algorithms for AutoIt. The images captured by the camera served not only for data recording but also required further processing. Conversely, other automation tasks —such as operating manual devices via buttons, executing pick-and-place operations (e.g., loading analytical instruments), or performing pipetting assays— entail substantially lower levels of automation complexity.

As pipetting is one of the most important processes in life science laboratories, we tested the capability of the approach in Rupp et al., 2024 (referenced as “robotic system” (RS)), automating two different pipetting assays. There, we also observed the pipetting precision in contrast to a manual user and a liquid handling station^19^.

### Flexible Localization

To apply LARS’ capabilities in any laboratory setting, several steps must be undertaken. First, the robot is moved to the desired location, such as a device or laboratory desk. All interacting devices must be within LARS’ range of movement and each must be equipped with a unique ArUco marker. To register the new position of interacting items, an AutoIt scan script enables the robot to observe its environment using the camera to search for specific ArUco marker IDs. Once LARS detects the position of the device, the initial poses of several corresponding device scripts are adjusted to the new position of the device. This procedure must be repeated whenever the position of LARS or the devices changes. If a new device is not yet automated, the device scripts must be programmed and saved for future automation. We provide a detailed description in supplementary file S1. The possibilities for automation with LARS depend on the application and the effort the user wishes to invest. As mentioned before, these can range from simple button presses and pipetting schemes to more complex processes, such as the pH value adjustment described above.

### Technical implementation

**Fig. 4:**
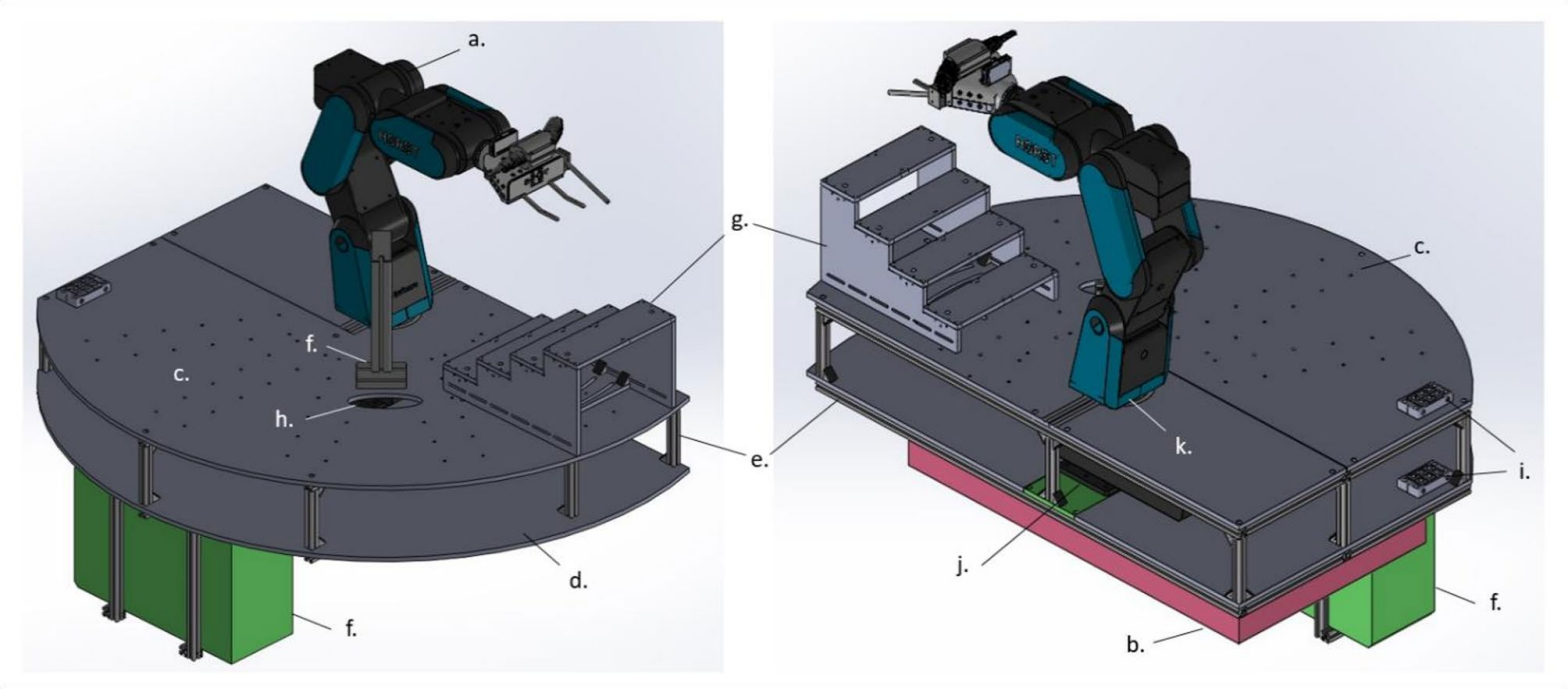
Accessory for hydraulic trolley. **a** 6-axis robot, **b** hydraulic trolley, **c** upper plate with hole pattern, **d** bottom plate, **e** aluminum profile, **f** switch cabinet, **g** stair hotel, **h** oval recess, **i** cable ducts, **j** robots’ feet, **k** axis 1, **l** stripping station.

For the mobile platform, we designed an accessory for a hydraulic trolley (see Fig. 4). This accessory consists of two plates, with the robot being mounted on the lower plate. The upper plate is attached above and enclosing the robots’ foot. The shape of the accessory allows the robot to utilize the entire range of the plate and to interact with devices/material on any laboratory desk. The inter plates space is intended for two purposes. First, to organize LARS’ cable management, and secondly, it offers space for the waste bag (pipette tips). The upper plate has a radially arranged hole pattern to allow fixed mounting of objects such as racks or devices. To enable tip exchange, we added a stripping station, which consists of a kind of V-shaped fork to drive in with the pipette and strip the tip by an upward movement of the pipette. Below the stripping station, we planned an oval recess in the upper plate in which the rejected tips are disposed of via waste bags. In addition, we added a stair hotel to create eight positions for microtiter plates, corresponding lids, reservoirs or tip boxes.

**Fig. 5:**
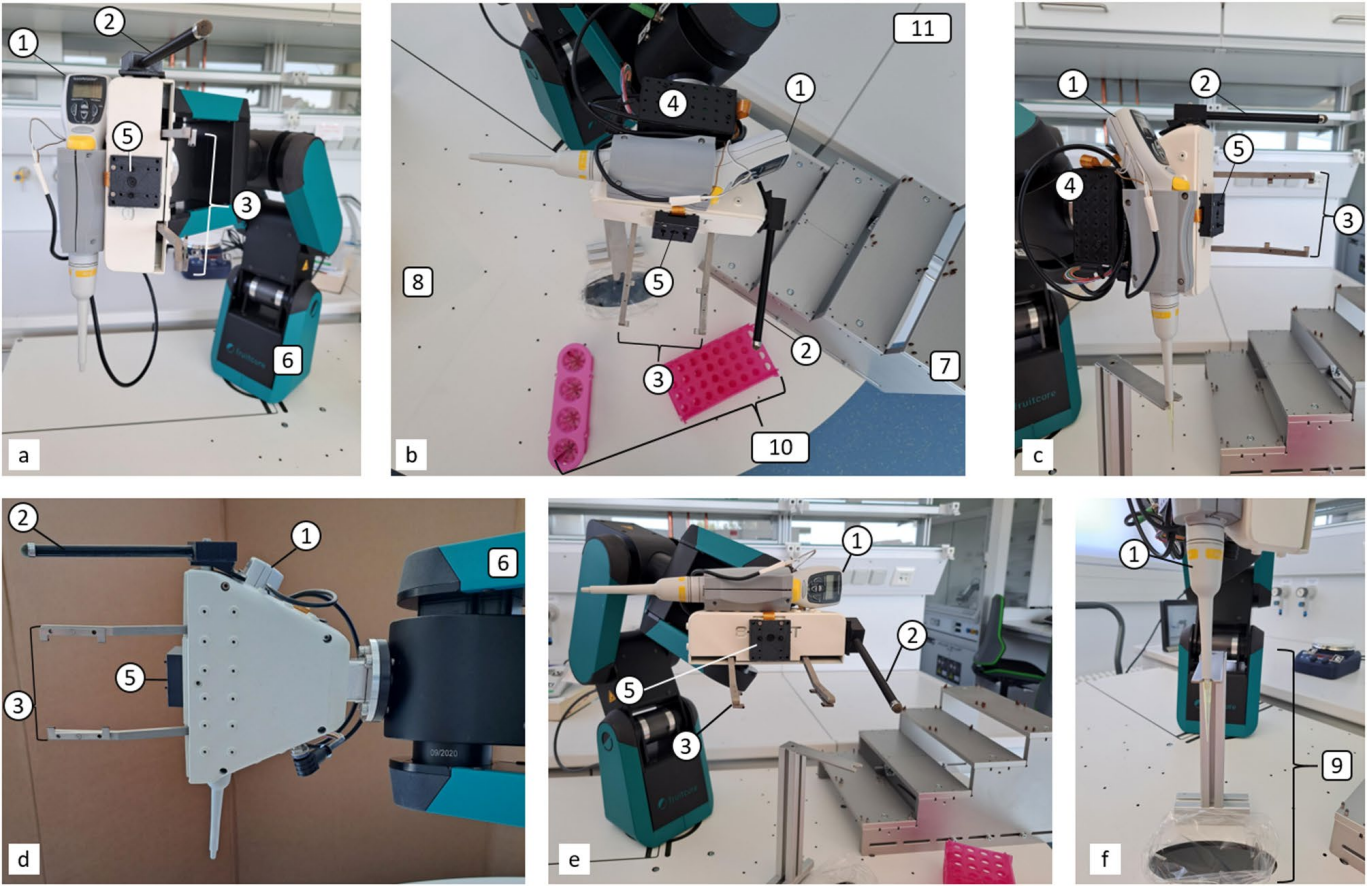
Detailed visualization of the end effector developed for LARS. The end effector is mainly used in two orientations: vertically for pipetting (**a, c, d, f**) or horizontal for plate gripping (**b, e**). The pipetting position is shown in a frontal view (**a**), side view (**c**) and view from below (**d**). The gripping position is shown in a top (**b**) and front (**e**) view. The end effector provides an electronic pipette (1) that is fixed on the top of the end effectors body. For rejecting pipette tips, we established a stripping station (**f**, 9). Next to the gripper (3), we provide an operating finger (2) for interaction with laboratory devices. The camera’s eye (5) is placed in between the two gripper jaws. We mounted the end effector’s functions control (Raspberry Pi, 4) behind the pipette. The end effector is mounted on a 6-axis robotic arm (6) that is encapsulated by the platform with perforated patterns (8). Through the perforated patterns, other objects such as a plate hotel (7) and diverse racks (10) can be mounted onto the plate. LARS enables the collaboration with other laboratory environments, such as a laboratory table (11).

For the “Swiss-Army-Knife”-principle based end effector, we completely redesigned 8-BOT’s labhand. We 3D printed a new case for the gripper with several threads to allow further assembly of components. For pipetting, we added an electronic single-channel pipette horizontally on the gripper. We integrated a camera eye into the end effector, thus it is positioned in the middle of the two gripper jaws. Next to the left gripper jaw, we mounted a 3D-printed operating finger with touchscreen compatible tip at the end. Therefore, digital (on touchscreen) and analogous buttons or switches on devices can be operated. A Raspberry Pi computer is fixed behind the pipette and controls the end effector. We provide a detailed visualization in Fig. 5 and a computer-aided design model as Supplementary Fig S1. The Raspberry Pi provides a web browser interface—called LARS CONTROL—that provides diverse options; initialize the gripper, open the gripper in landscape and portrait size, close the gripper and trigger the pipette (for aspirating or dispensing). In addition, the camera functions are available on this interface. Using the ArUco marker’s ID, the translation and rotation coordinates—as relative distance between the camera’s eye and the centre of the specific marker—are provided at LARS CONTROL. The interface of LARS CONTROL is shown in the Supplementary Fig. S2.

As previously described, we deployed a Windows PC as the main control centre with an automated Windows user controlled by AutoIt scripting routines. AutoIt interacts on the one hand with the Linux HMI of the robot (via TightVNC) and on the other hand, operates LARS CONTROL via web browser (provided by the Raspberry Pi). To allow automated processes, we utilized diverse AutoIt scripts to fulfil different procedures. Thus, AutoIt controls the robot control software HorstFX, meaning starting and closing specific robot programs, interacting with the end effectors interface and calculating relative robot coordinates that depend on the cameras provided information. We utilized the camera to locate laboratory devices. Therefore we used ArUco markers that are placed on the laboratory devices, as previously described in Wolf et al. 2023 with the principle described by Sani and Karimian, 2017^20,21^. In addition, AutoIt is capable to communicate with users via GUI, as shown in the pH adjustment example. As virtual Windows-User, AutoIt is able to control any Windows-based software application. Therefore, further software (e.g., analytical device software) can be integrated into LARS. LARS is also compatible with the Python image read out program^18^ for digitalization of laboratory display values. Our previously published Voice-User-Interface is compatible with LARS and can be used as an alternative way to control the system^22^.

### General evaluation of LARS

LARS with its three components—the platform, the robot with the multifunctional end effector and the software automation by AutoIt—seems to provide useful features for smaller bio(analytical) (research) laboratories. To target this group of laboratories, we especially tried to fulfil the requirement of flexibility and to keep automation as simple as possible. Certainly, more advanced technologies are already established—for example mobile, self-driving robots are no longer a novelty—but such technologies are neither technically nor financially feasible for this type of organization^23^. As another guiding principle during development, we decided to avoid in-house designs (e.g. tube holders, tip holders or similar) as far as possible but instead to integrate existing objects of a random laboratory. We have observed that this additional equipment quickly drives up the purchase price of supposedly inexpensive commercial systems.

With LARS, we were able to considerably expand the potential for future exploration in the field of flexible automation suitable for smaller laboratories. As a proof-of-principle, we presented how laboratory automation can be realized with only one system, automating as much laboratory equipment as possible. Therefore, our strategy tries to imitate a laboratory operator, meaning LARS is capable of automating devices regardless their conditions (e.g., if they support SiLA). This includes also the ability to read-out displays of laboratory devices. Also, the possibility of integrating our AutoIt-based voice assistant shows the possibility of equipping LARS with additional functions on the software side^22^. Due to the design of the platform, we offer users the option of automating long-term, fixed positions on the platform itself—or integrating devices in the laboratory environment at shorter term via the ArUco markers. Thus, LARS can be moved between different positions and laboratories manually, for interacting with diverse laboratory environments. We are also striving to optimize the end effector. A miniaturization regarding the size of the end effector would be helpful to improve the range and agility of the robot. For the gripper, we think about extending the functionality to realize the transport of smaller (and round) vessels. In addition, we are working on uncapping of screw caps (e.g., 15 mL and 50 mL falcons). We have already tested a method for opening micro reaction vessels using a 3D-printed “bottle opener” (modified according to WaveSupportApparatus, 2017^24^) by fixing the mechanism to the operating finger. LARS generally requires more time for both spatial orientation (via ArUco markers) and automated processes (e.g., pH adjustment) in comparison to a human performing the same tasks. Nevertheless, these strategies significantly reduce the workload for laboratory personnel. Our strategy of exclusively automating existing devices failed with centrifuges. It is a well-known fact that there are special centrifuges in automation that differ slightly from the principle of conventional centrifuges. The rotor lid, balancing samples (uniform distribution) and the identification of samples after centrifugation, for example, represent problems for automation via LARS. LARS also shows limitations in aseptic operations (microbiology or cell biology). While smaller robotic arms have proven their suitability for aseptic applications, LARS needs to operate outside the laminar flow bench due to its dimensions^25^. As a result, the radius of action would be extremely limited, thereby making automated processes with LARS impossible. Nevertheless, even in such laboratories, LARS is able to improve laboratory routines—an example is shown here with the pH value adjustment of buffers. We also want to highlight, that automation is usually especially beneficial for repetitive tasks, as this is the supreme discipline for automated systems such as robotic arms. In the range of small and medium sized laboratories, automating such processes might have a high impact as laboratory operators are able to focus meanwhile on other—more complex—tasks^26^. The automation efforts should not focus on maximizing the number of automated tasks, but on targeting repetitive and time-intensive processes, as these contribute significantly to operator fatigue and are associated with a higher risk for human error.

## Outlook

Despite the quality advantages especially in bioanalytics, the automation of laboratory processes in smaller bioscientific (research) laboratories is still reserved. Commercial solutions are usually inflexible, complex or too expensive. In this paper, we have directly addressed the unique needs of these laboratories by developing a strategy that we have successfully implemented with a prototype. The results of this strategy exhibit remarkable versatility, offering significant benefits across a broad spectrum of research and industrial processes in smaller laboratories. As LARS operates similarly to a laboratory operator, existing devices can be easily automated beyond manufacturer boundaries. The mobility of the system in combination with the multifunctional end effector enable the automation of a wide range of applications in the bioscientific environment. In the future, enhancements to the robot’s agility and the end effector’s "Swiss-army-knife" concept are anticipated, expanding its ability to manage various types of containers. LARS has already demonstrated substantial flexibility during this project phase. We assert that our strategy holds significant potential and that our prototype represents a foundational advancement. It establishes a highly autonomous system capable of meeting the diverse needs of a wide array of laboratories in the life sciences sector.

## Electronic supplementary material

Below is the link to the electronic supplementary material.

Supplementary Material 1

Supplementary Material 2

## Data Availability

The datasets generated during and/or analysed during the current study are available from the corresponding author on reasonable request.
