# Supplementary material for: A mobile robot bridging manual and automated bioscientific workflows by applying the Swiss army knife principle
Source: Sci Rep. 2025 Jun 20;15:20160. doi: 10.1038/s41598-025-05404-3 (PMC12181227; doi:10.1038/s41598-025-05404-3)
Supplement: Supplementary file 1 — Supplementary Material 1 [file 41598_2025_5404_MOESM1_ESM.pdf]

Link to the supplementary video S1

Video S1. Experimental application of LARS. Automation of the buffer pH value adjustment process with LARS’s capabilities. The video shows the whole automated process.

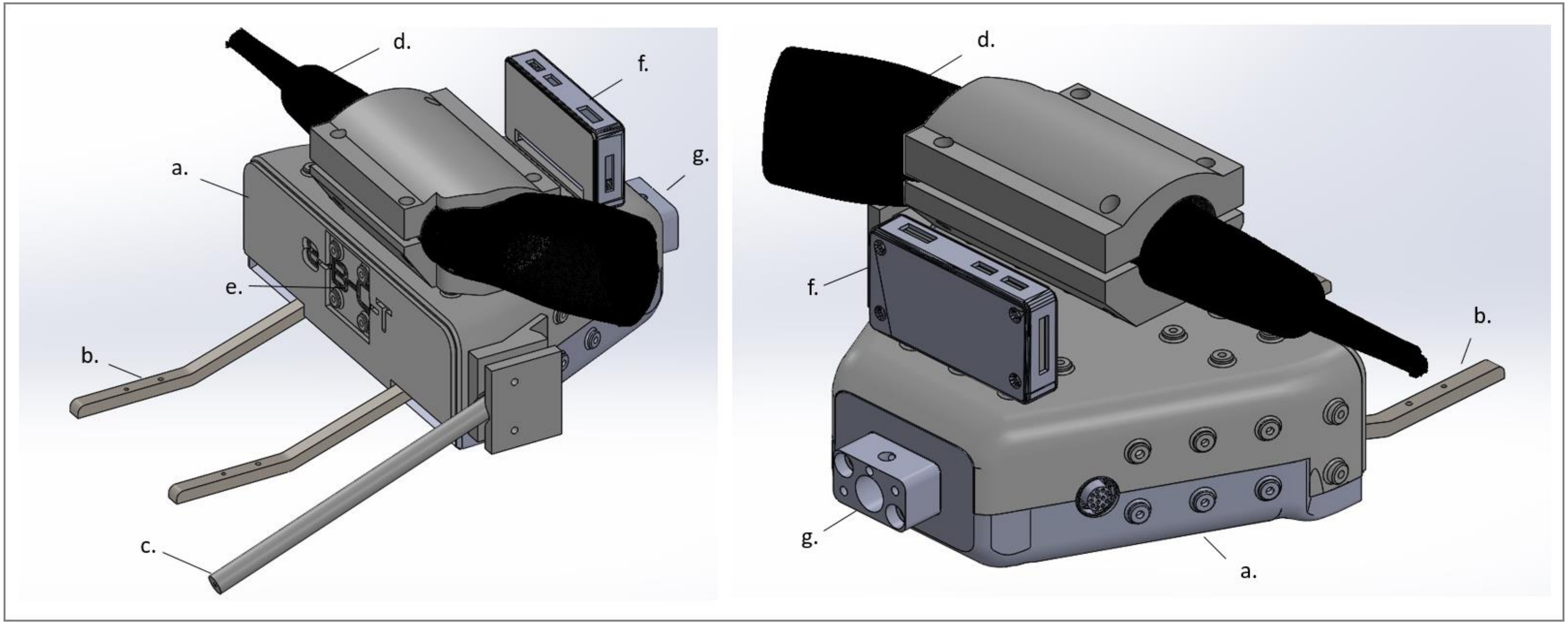

Figure S1. Multifunctional end effector for biomedical laboratory approach. **a** 8-bot gripper as basis with **b** gripper jaws for Wellplates. **c** The operating finger is used for operating switches and buttons of laboratory devices. **d** A Pipette is mounted horizontally, end effector rotates by 90° to allow automated pipetting. **e** A camera, for object recognition is integrated into the main body of the end effector. **f** A Raspberry Pi is mounted and programmed to control the end effectors functions by providing a browser web interface called LARS CONTROL. **g** A connection carrier enables the end effector to be mounted on any robot flange.

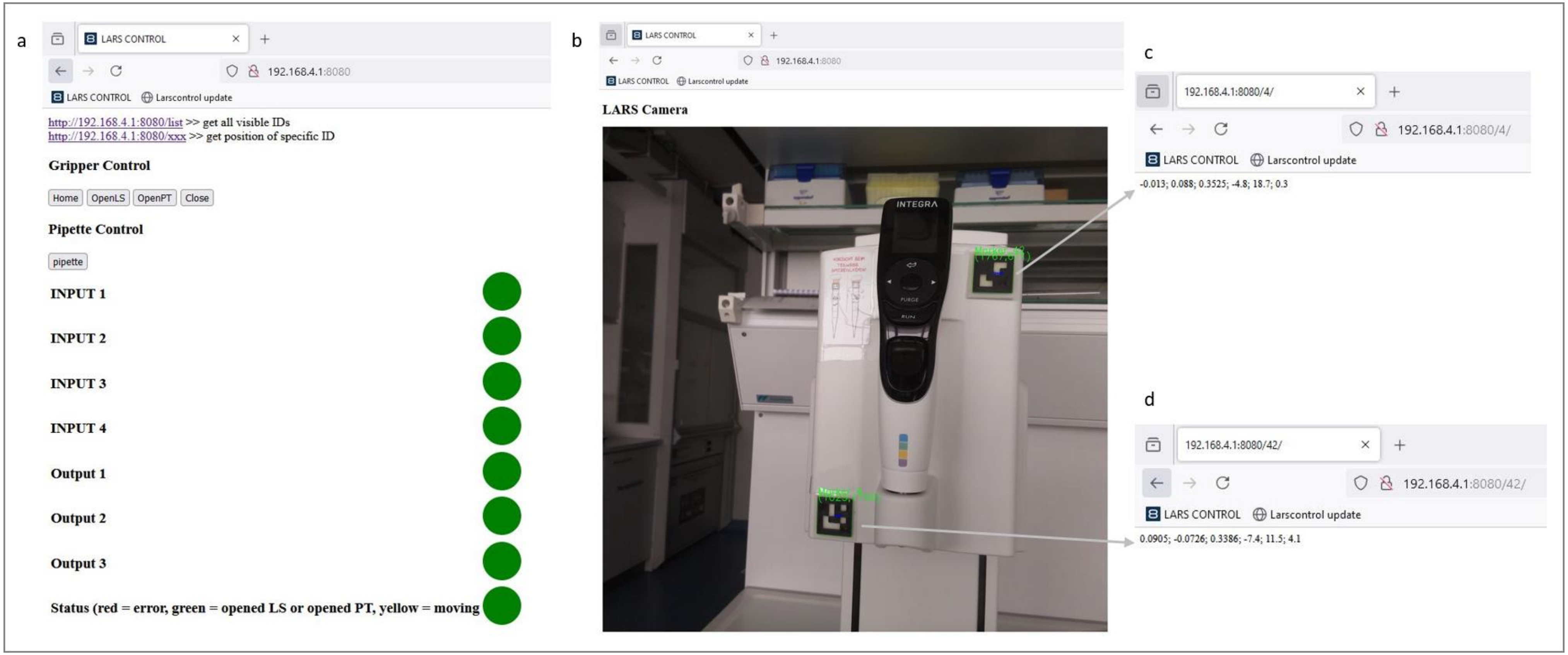

Figure S2. User interface of the multifunctional end effector. **a** The connection between Windows PC and LARS CONTROL can be started via the browser interface. Buttons are available to initialize the gripper, to open it in landscape or portrait and to close the gripper. Another button triggers the pipette function. The green dots indicate the status of the end effector and change the colors at times. **b** The interface displays the current image of the camera. If an ArUco marker is visible, the system adds the ArUco marker ID and their coordinates related next to the marker on the image. **c,d** To get the coordinates of an ArUco marker, the user has to add the ArUco markers ID in the address commando line. The coordinates are provided as relative distance from the camera eye to the marker in meters (x, y, z, rx, ry, rz).

Integrating new devices

As previously mentioned, we used fiducial markers (ArUco Marker) as common for laboratory devices detection using mobile robots [1]. Therefore, we programmed a detection routine in AutoIt that initiate the robot to scan its environment, searching for a specific marker ID on a corresponding device. AutoIt stops the robot's scan immediately as soon as the marker is detected by the camera. Then, AutoIt obtains the relative translation and rotation coordinates by the end effector's web interface. In a further step, AutoIt calculates and writes a robot script to moves the robot in to an 'initial pose' (with the camera eye as Tool Centre Point (TCP)). This initial pose is a frontal pose in 250 mm distance to the marker (resulting in relative camera coordinates x=0; y=0; z=0.25; rx=0; ry=0; rz=0 given in meters), that serves as reference pose for all actions concerning the specific device. This means, operating a device with LARS requires a separate HorstFx script for each action in relative movement. For example, a particular plate shaker features four different HorstFx scripts: one for the shake function switch, one for the timer function switch, one to position the plate and one to retrieve the plate. All those HorstFx device scripts consists of relative movements depending on the start position that is the absolute (current) initial pose. Consequently, AutoIt saves the coordinates of the current (initial) position of the robot as start point in all relating HorstFx-scripts. To integrate new devices or new actions on devices, the robot must be moved to the specific initial position by AutoIt's "Scan" script, specifying the unique ArUco marker ID. Then the user has to save relative movement points for the specific action and register the HorstFx script names into AutoIt. Figure 1 shows the schematic procedure of detecting a laboratory device with LARS.

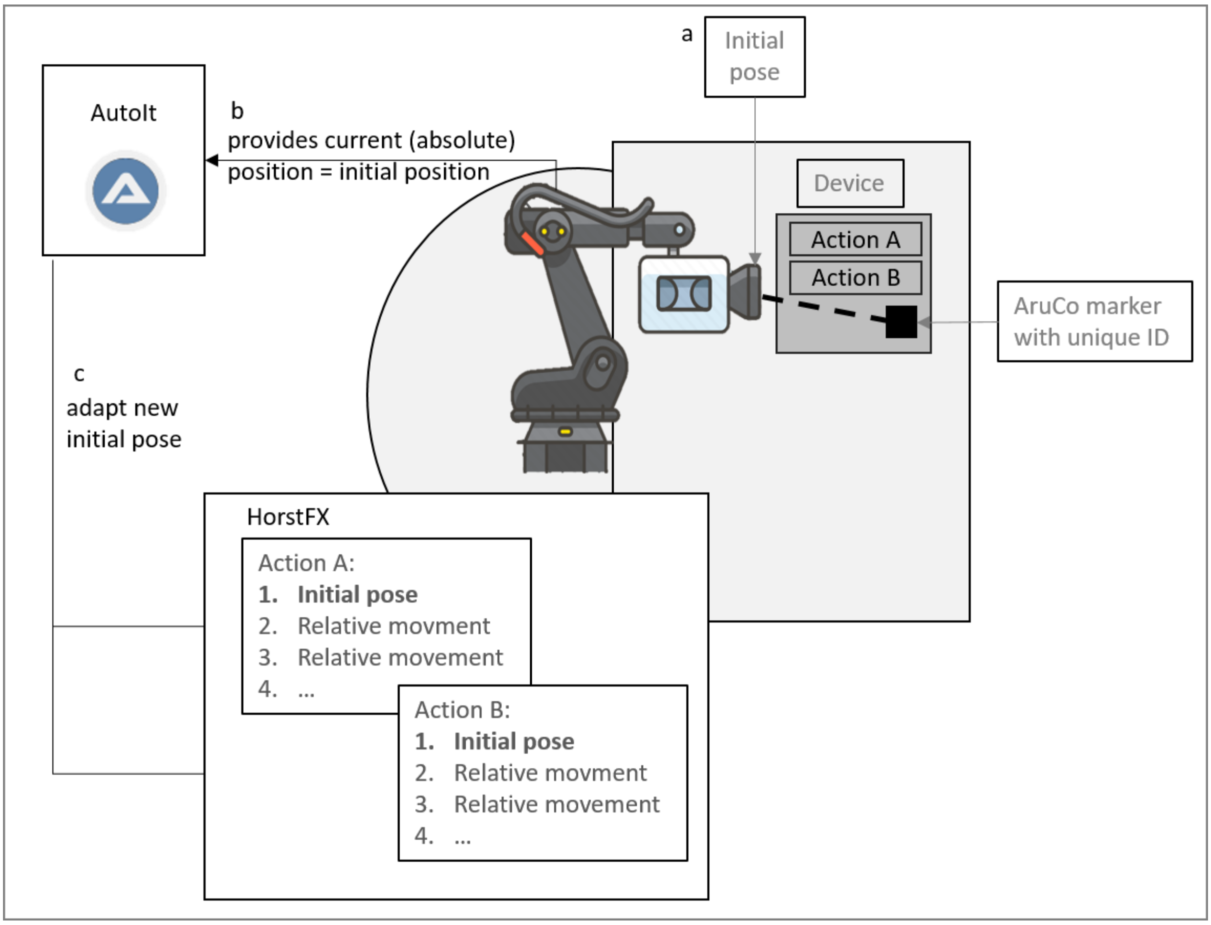

Fig. 1. Integration of new laboratory device position. After the robot detects an ArUco marker of a specific device, AutoIt calculates and moves the robot to the corresponding initial position **a**. This initial position (=current position) is provided as absolute coordinates by HorstFx **b**. AutoIt adapt these coordinates into all device related HorstFx scripts **c**. There, the initial pose acts as start position for an action on the device that is programmed as relative movement.

References

1. Wolf, Á. *et al.* Towards robotic laboratory automation Plug & Play: The “LAPP” framework. *SLAS technology* **27,** 18–25 (2022).
